# Protective and Modifying Powers of Vaccination

**Published:** 1857-01

**Authors:** Edward C. Seaton

**Affiliations:** Vice-President of the Western Medical and Surgical Society of London.


### ORIGINAL COMMUNICATIONS.

### ON THE PROTECTIVE AND MODIFYING POWERS OF VACCINATION.

By EDWARD C. SEATON, M.D., Vice-President of the Western Medical and Surgical Society of London.

[*Read at the First Meeting of the Society for the Session* 1856-7.]

FIFTY-EIGHT years have now passed since the first promulgation to the world of that great discovery of Jenner, which has already saved so many millions of human lives, has rescued so many millions more from hideous deformity, and has justly procured for its author a place among the most illustrious benefactors of mankind. In his first treatise, entitled *An Inquiry into the Causes and Effects of the Variolæ Vaccinæ*, published in 1798, Jenner gave a lucid account of the disease popularly known as the cow-pox; showed how it was frequently communicated by accident to man, and detailed the phenomena produced by such communication; gave proof, by long continued observation and by experiment, that when the system had been thus once infected, it remained afterwards secure from the infection of small-pox, whether by variolous effluvia or by inoculation; showed that the cow-pox itself might be propagated from man to man by inoculation, and that so propagated it exercised the same protective power as when taken directly from the cow; and suggested, therefore, that it might be possible to introduce a mode of inoculation safer and in every respect preferable to the variolous inoculation, which was then in use. "This inquiry", he modestly says in conclusion, "I shall myself continue to prosecute, encouraged by the hope of its becoming essentially beneficial to mankind." To the further prosecution of this subject it is well known that he devoted the remainder of his life. The progress of his experiments, observations, and opinions, may be traced in his various works, and in his published letters: and it is a striking proof of his sagacity, and of the profound thought he had given to the subject, that his rules and cautions with regard to the process of vaccine inoculation, or vaccination, as it is now termed, are those which guide the practice of the best vaccinators of the present day; that his theoretical view of the identity of cow-pox with human small-pox has received decisive proof, in our own time, from the experiments of Ceely; and that his sanguine anticipations of the benefits to be conferred on mankind by his discovery are far on the way to be realised,—if not absolutely and entirely, at all events to all practical purposes.

To say, indeed, that he was in error in none of the views which he propounded, or that he left nothing to be added to his discovery, would be to claim for him foresight more than human. But these errors and deficiencies were few ; the more his writings are studied, the fewer will they appear to be ; and, in many instances, where his statements have been called in question, it has happened that particular expressions have been taken hold of, and used in a sense in which he certainly never intended them. When he spoke, for example, of the security against small-pox afforded by vaccination as *perfect,* he meant (and by his very hypothesis of the identity of cow-pox with small-pox he could have meant nothing else) that it would protect the constitution to the same extent and in the same way in which an attack of variola itself would. "Duly and efficiently performed", he observes, "it will protect the constitution from subsequent attacks of small-pox as much as that disease itself will. I never expected it would do more; and it will not, I believe, do less." Whether this opinion is sustained by all the facts which have now accumulated, or whether, indeed, there are yet facts enough of a precise and positive character to enable us to determine the question authoritatively, is one of the points we shall have to enter upon immediately ; but the opinion is clearly something very different from that which has been attributed to him, viz., that he represented vaccination as an *infallible preventive,* and maintained the *impossibility* of small-pox after it.

Again, when he looked forward, as he undoubtedly did, to the extermination of small-pox by vaccination, it is not to be supposed that he expected such a consummation to be brought about until the practice should have become universal : and when this shall be so, as it will certainly one day, however tardily, be, we shall have to see whether his expectations may not yet be realised. The charge of presumptuousness which has been brought against him may then have to be withdrawn ; and the note of triumph, which was sounded a few years ago, because after a lapse of fifty years small-pox was still rife amongst us, may be changed. Not fifty, nor five hundred years, will suffice to determine the truth of the anticipation, while there remain any unprotected to receive and to convey the seeds of that pestilent disease.

It is greatly to be lamented that the rules and precautions which Jenner laid down with so much care and precision, for the performance of vaccination, should have been in so many instances neglected or departed from; and that, from the very simplicity of the operation, it should not always have received, either from medical men or from patients, that attention to which it was entitled, as an operation intended to protect for life from the attacks of a fearful disease. Had this not been so, we should at all events have been free from one difficulty, which encounters us now at every turn, in our endeavours to estimate the exact protective value of the Jennerian discovery,—that arising, namely, from the necessity of determining, in cases in which vaccination is said to have failed, whether there has really been effective vaccination or not. In such a society as this, I need scarcely say that it is not the insertion of lymph into the arm, nor the production merely of a vesicle, which constitutes vaccination: *the vesicles to be produced must have a specific character, and go through a definite course, indicative of a particular constitutional affection: and it is only when this character is perfect, and this definite course has been normally gone through, that there is protection;* or at all events a full measure of protection. Of inefficient and spurious vaccination there was plenty in Jenner's time, and he taught and wrote much about it; and we have it on the testimony of men from all parts of the country, largely engaged in vaccinating, that there is plenty still. This is a point to which I shall have again to advert hereafter.

That efficient vaccination will protect the system against an attack of small-pox absolutely as a rule, and that even under circumstances of the severest exposure, is so well known that it needs no proof nor illustration. It is equally certain that small-pox will sometimes occur after the most perfect vaccination; that it will occur with much greater frequency after spurious or imperfect vaccination; and that it may be met with in those who have already had the disease casually or by inoculation. But when we strive to determine and compare the ratio in which individuals, protected by vaccination, perfect or imperfect, and those protected by casual or by inoculated small-pox, are liable to take small-pox subsequently, we find the greatest possible difficulty. For this comparison we need, in the first place, that the inquiry should be made on a sufficient number of individuals similarly circumstanced as to age, condition of life, various external circumstances, and liability to exposure: where vaccination is alleged, we have, as I have already said, to determine whether there has been real vaccination or not: and where small-pox is alleged, we have to determine that it was this disease which really had been gone through: then, as regards the alleged small-pox occurring after such vaccination or previous small-pox, we have to be certain that there is no error in diagnosis,—that we have really before us a case of variola, and not one of those many affections which have at times been mistaken for it. Now it is obvious that such an inquiry as this can only be instituted with regard to particular classes of individuals; and as regards one class of individuals—children under puberty—the inquiry *has* been made under circumstances which admit of every confidence being placed in the result.* Every boy admitted into the Royal Military Asylum at Chelsea, not bearing satisfactory marks of small-pox or cow-pox, is vaccinated; and we get thus a community, all of them protected, but some by inoculation or casual small-pox and some by vaccination. Now, of 5,774 boys admitted from the opening of the establishment in 1803, to December 31, 1851, 1,950 had on admission marks of small-pox, and 3,824 had either marks of vaccination, or were on admission vaccinated. Of the 1,950, twelve, or 6.15 per 1,000, had small-pox subsequently; of the 3,824, twenty-seven, or 7.06 per 1,000, had small-pox subsequently. Now here we had present all the conditions to which I have referred as requisite for a perfect comparison: and not only the number of persons, but the length of time over which the observations extend, warrant, as Dr. Graham Balfour justly says, reliance being placed on the results; and these are, that as regards children under puberty there is scarcely any difference in protective value between cow-pox and previous small-pox, and so far the opinion of Jenner is entirely confirmed. And, before going further, I must remark that the experience of this asylum places beyond all doubt that the recurrence of small-pox, or its occurrence after inoculation, is by no means the very rare thing it has been represented by some to be. We read statements of a vague estimate by De La Condamine that the cases which so occur are not more than one in 10,000, and we are even told to look upon this as exaggetated; but we have here an example in which out of every 1,000 more than six have had a second attack in a population certainly not peculiarly exposed to infection, and before any

---

* Dr. T. G. Balfour, On the Protection against Small-Pox afforded by Vaccination, in *Medico Chirurgical Transactions*, vol. xxxv.

have reached the age of manhood. I have already adverted to the care which must be taken in investigating such cases,—as indeed in investigating cases of small-pox after alleged vaccination; but in this instance the evidence of the primary disease was, in every case, the characteristic marks left by it, and the second attack was watched through its whole course by observers whose competence to discriminate accurately cannot be called in question. And I must express here emphatically my conviction that, if a case of small-pox has been observed *throughout its course* by a medical man, there is not often a mistake. In an admirable paper by Mr. Marson,* to which I shall have occasion to refer by and bye, he mentions that 185 cases have been sent to the Small Pox Hospital in sixteen years as variola, which turned out to be no variola at all;—but these, it must be remembered, were all or mostly sent in the onset of the disease, when the difficulty of diagnosis must be acknowledged by all. The limits of this paper do not allow me to pursue this subject further, nor to present a variety of facts confirmatory of the view I have advanced, that small-pox after small-pox may occur more frequently than has been supposed: but it was essential to draw attention to the subject, which must be well kept in mind in any review we may take of facts bearing on the protective value of vaccination.

The comparison we have made in the case of the Royal Military Asylum between the protection of vaccination and that of previous small-pox is complete as far as it goes; but it unfortunately stops short just as we are entering the period of life when the protection of vaccination is said by some to begin to wear out, that is to say, the age of puberty. Now the army gives us a class of men, all of whom have passed this period of life, and all of whom by the rules of the service, should be, and we presume are, protected. About 78 per cent. of the whole are protected by vaccination, and the remaining 22 per cent. by previous small-pox. Now, in a force so protected and in many instances, as Dr. Balfour has shown, considerably exposed to small-pox, the annual ratio of cases has not been greater than sixty-six to every 100,000 men, or rather more than half a case per 1,000 men. Unfortunately these statistics are not available for the comparison we desire to institute of the *relative* protection afforded by vaccination and by previous small-pox, because it is not recorded with regard to the cases how many occurred in

* On Small-Pox and Vaccination. *Medico-Chirurgical Trans.*, vol. xxxvi.

those protected in the one, and how many in those protected in the other way. But they are unexceptionable evidence of the general protecting value of vaccination, four-fifths of the force owing its protection to that alone.

In the navy, by the rules of which service also all should be protected, there is the same impossibility of ascertaining how many cases have occurred in the vaccinated and how many in those protected by previous small-pox: and there is also the same general testimony to the protecting value of vaccination. In this force the cases are slightly more numerous, having been 417 on an aggregate strength of 363,370 men, or rather more than 1 (1.148) per 1,000. This increase over the ratio prevailing in the army (115 per 100,000 as against 66 per 100,000) Dr. Balfour attributes to the crowding on shipboard, and the consequent difficulty of separating the healthy from the sick. Doubtless these causes would so operate; but on the other hand it is probable that sailors, though often considerably exposed, as Dr. Balfour has shown, are less so on the whole than soldiers, who are frequently quartered for long periods together in large towns: and I do not doubt that the real explanation is that the rules of the service are not so well carried out, for many obvious reasons, amongst them as in the army. In looking over the returns from the particular ships in which small-pox had appeared, I find it stated expressly in some cases that there had been *no* protection, and in others that it is doubtful whether there had been protection or not.*

To understand the full value of these facts it is desirable to compare them with the results ascertained in the Royal Military Asylum, and to make the calculation therefore for that institution on the same principle on which it is made for the army and navy, that is, according to the aggregate strength and not according to the mere numbers admitted: it will then appear that the proportion of attacks from small-pox for every 100,000 individuals is

| | |
|---|---|
| Among the soldiers - - - - - | 66 |
| Among the sailors - - - - - | 115 |
| Among the boys - - - - - | 123 |

The immunity therefore is absolutely greater among the soldiers and sailors than it is among the boys. It is quite true that the relative protection afforded by vaccination and

* Even in the army it is probable there are some unprotected, especially on colonial stations. Inspector-General Dr. John Davy states that it is only by the strictest superintendence, and by returns at short intervals, that thorough vaccination can be kept up.

by previous small-pox may not be the same, as it has been ascertained to be among these latter ; we have no facts enabling us to state whether it is so, or whether it is not: but as to the wonderful extent of protection afforded by vaccination in classes quite as much exposed to small-pox as the bulk of the community, and the individuals comprising which are above the age of puberty, the returns are conclusive. I may remark too that the great majority of the cases in the army occurred between the ages of fifteen and twenty-five, a period of known proclivity to small-pox; and that there were comparatively few after this latter age, a result quite at variance with the notion that the vaccine protection gradually wears out,—for, on that hypothesis, the longer the period that has elapsed since vaccination the greater should be the liability to attack. Now, in the United Kingdom, in ten years, there were among the soldiers forty-three deaths from small-pox on an aggregate strength of 133,874 under twenty-five years of age, and only twelve deaths on an aggregate strength of 110,723 above that age. This question is one of the greatest importance, and demands a separate investigation, into which I hope at some future time to enter. In the meantime, I will only observe that I am in possession of a large number of facts from returns to the Epidemiological Society, which are quite in accordance with this result, and which show that the liability to small-pox after twenty-five in persons vaccinated is far from increasing, as it should do if the vaccination wore out.

But it is desirable to subject vaccination to a severer test, and to inquire what is the degree of immunity afforded by it under circumstances of long continued and frequent exposure during epidemic influence. These are the conditions under which the greater number of failures undoubtedly occur, and they are also those under which the practice has to record its greatest triumphs, for it is precisely under these conditions that without protection *scarcely one* escapes. There are not observations sufficiently accurate and on a sufficiently large scale to justify positive numerical conclusions on this subject: but some interesting facts have been observed. During an epidemic in Chelsea in 1838-9,* Mr. Marshall inquired into the circumstances affecting 757 individuals, all of them severely exposed to small-pox, for they were members of families in each of which there had been at least one case (generally a fatal one); and in the condition of life in which they were, a case in the family implied in almost

---

* *Lancet*, vol. xxxvi.

every instance a case in the room which they inhabited. Of the 757 there were only 231 reputed to be vaccinated ;* there were no means of ascertaining with regard to these the character of the vaccination, but of the whole number twenty-seven only suffered from small-pox, while of the remainder every one, who had not had previous variola, had small-pox on the occasion of this epidemic, except seven. Unfortunately Mr. Marshall did not inquire, at all events he does not state, how many had been protected by previous variola, but he mentions distinctly that there were fourteen cases of secondary small-pox. For want of this inquiry, however, his return is not available for a rigorous comparison of the relative protection afforded by variola and vaccination under these circumstances of severe exposure. It occurred to the Epidemiological Society some time ago, that valuable information might be acquired on this point by ascertaining the protection, which medical men themselves experienced from these two methods,—largely exposed as they must be to the sources of infection. Accordingly two series of inquiries were issued with the view of ascertaining this. The analysis of the second and larger series is not in such a state as enables me to make use of it on the present occasion ; but with regard to the first these are the results. Out of 347 medical men protected by vaccination there were 44, or 12.6 per cent., who had had variola subsequently, and out of 82 who had been inoculated in their infancy there were 3, or 3.6 per cent. who had subsequently small-pox. It is not for a moment to be supposed that these numbers represent the percentage in which medical men, in the ordinary practice of their vocation, are subject to variola. For, in the first place, a large number of those to whom the questions were addressed made no reply at all : now as the occurrence of variola, whether after a previous attack or after vaccination, is undoubtedly the exception, the probability is that all who had so suffered would reply, and that, conversely, of those who did not reply few or none had suffered,—and this would materially alter the percentage. In the next place, several of the cases were taken in the dissecting-room, and not in the course of practice at all ; and, lastly, it must be remembered that the persons to whom the inquiries were sent were *selected* persons, and many of them selected because of the known extent to which they were in the habit of meeting with the disease. But for comparison between the relative protective

* A remarkable proof of the extent to which vaccination was at that time glected.

powers of variola and vaccinia this return is of great value, and conclusions drawn from it may be relied on, so far as any conclusion can be relied on, which is made on so limited a number of observations: and it would appear, putting aside any reference to the special character of the vaccination, that the protective power of small-pox is considerably greater than, numerically three and a half times as great as, that of vaccination under these circumstances of great exposure. But we must bear in mind the observations that have been made with regard to efficient and inefficient vaccination; and an analysis of these cases, with the view to determine the character of the vaccination in each of them, will give us some interesting results.

Of the 347 persons there were, having

| | Number. | Attacked. | Per Cent. |
|---|---|---|---|
| No cicatrix visible | 18 | 3 | 16·6 |
| One cicatrix | 62 | 7 | 14·0 |
| Two cicatrices | 173 | 26 | |
| Three cicatrices | 36 | 1 | 3·2 |
| Four cicatrices | 20 | 1 | |
| Six cicatrices | 6 | 0 | |
| No mention | 32 | 6 | |

Of those in whom the cicatrices are mentioned as good, there were with

| | Number. | Attacked. | Per Cent. |
|---|---|---|---|
| One cicatrix | 48 | 5 | 12·0 |
| Two cicatrices | 158 | 21 | |
| Three cicatrices | 34 | 1 | 3·5 |
| Four cicatrices | 17 | 1 | |
| Six cicatrices | 6 | 0 | |

Now, taking these facts in connection with the immense value of the cicatrices as an index of the efficiency of vaccination, shown by the researches of Mr. Marson to which I shall allude almost immediately, they are certainly sufficiently remarkable: so far as they may be trusted to, the liability to take small-pox, in persons having more than two cicatrices of vaccination, is no greater than to take small-pox after previous small-pox: and if the observations are too few, as they undoubtedly are, to justify our drawing any positive conclusion to this effect, they require us at least to pause and to call for more facts before we acquiesce in an opposite conclusion, or admit that, duly and efficiently performed, vaccination will not protect the system, as Jenner stated, to the same extent that inoculation itself would have done.

It is a confirmation of this, that, during seventeen years, not one of the servants or nurses of the Small Pox Hospital has been attacked by small-pox, though vaccination has been the only protection of many of them.

I have endeavoured, as I have gone along, to make sufficiently clear the conclusions, which I think we may draw from the foregoing facts, with regard to the protecting power of vaccination: but I may be allowed briefly to recapitulate.

1. It would seem that there is no important difference between the protecting power of variola and vaccinia during childhood, under circumstances of ordinary exposure: with regard to severe exposure, there are not facts to determine one way or the other.

2. There are not facts to determine the relative protecting power of variola and vaccination in adults under ordinary exposure; but there are abundant proofs of the enormous amount of protection afforded by vaccinia.

3. Adults severely exposed, relying on what is ordinarily termed vaccination, will probably take small-pox, though, as we shall see immediately, of a modified kind, in a greater ratio than those having previously had small-pox, by inoculation: but

4. If the vaccination has been thorough and efficient, it is extremely probable that the liability to small-pox under severe exposure is no greater than after inoculation.

5. Those statements, therefore, are entirely without foundation, which speak of small-pox after inoculation as a risk hardly exceeding a possibility, and never to be taken into account: while small-pox after vaccination is represented as a thing of daily and constant occurrence.

6. The representation that the protection of vaccination gradually wears out, till at length it leaves the system as liable to attack as though the protection had never been imparted, is not only unproved, but is opposed to important facts, and in all probability will turn out to be unfounded.

7. There is, however, a great proclivity to small-pox, whether natural or after vaccination, between the ages of fifteen and twenty-five,—a circumstance which will account for many of the facts, which have been cited as proof of the non-durability of the vaccine protective power.

The remarkable power of vaccination in modifying the course of an attack of small-pox, and in thus diminishing the fatality of the disease, is well known to all, and admits of being estimated with very considerable precision. Of 2,654 cases of natural small-pox admitted into the Small Pox Hospital in sixteen years, in only sixty-nine was the eruption irregular or modified, and of the remaining 2,585, in 1,821 it was confluent; while of 3,094 cases reputed to be vaccinated it was modified in 2,149, and of the 945, in which the course was unmodified, there were but 570 confluent cases, 138 of which were in persons simply stated to have been vaccinated, but having no mark whatever, and 275 in persons having but one cicatrix. In various epidemics recorded by writers, and in others communicated to the Epidemiological Society, the result has been the same. With the view of showing at a glance the effect this modification has on the mortality of the disease, I have thrown into a table the results of observations in various epidemics at home and abroad; the results of returns made to the Epidemiological Society by practitioners residing in different parts of the country, who have kept numerical records of their cases of variola; and the observations of the Small Pox Hospital. (See next page.)

On this table I have only a few remarks to make. The returns to the Epidemiological Society will be found to present a favourable view, both as regards the mortality of natural small-pox and of small-pox after vaccination. They are from practitioners in the country as well as in towns; they include sporadic as well as epidemic cases; they do not, indeed, comprehend any *severe* epidemic in a large town; they include comparatively few hospital cases, but cases in workhouses are of course comprehended. It will be seen, then, that they are, to a great extent, free from two causes which so greatly aggravate mortality: epidemic influence and hospital aggregation. The deaths from small-pox after vaccination throughout the table are deaths after *reputed* vaccination. The statements are given as reported, and could they have been thoroughly investigated, it would doubtless appear in many instances that the evidence of vaccination was unsatisfactory: indeed, in several of the cases in which death occurred, this want of evidence is mentioned by the reporters themselves, and the same, I may remark, is the case with regard to the epidemics recorded, which stand distinct in the table from the society's returns. In the epidemic in the Mauritius, for example, where thirty deaths are recorded among 421 cases, it is expressly stated by Mr. Gardner, that in *thirteen of these deaths only* were the evidences of vaccination satisfactory; and in the returns from the Small Pox Hospital and from Ceylon the evidence of vaccination has been thoroughly investigated with very remarkable results, as we shall immediately see. The returns, however, given in the table from these sources are after *reputed* vaccination, as in the other cases. Another mode in which this table exhibits results too unfavourable to vaccination is, that except

| | Natural Small-Pox. | | | Secondary Small-Pox. | | | Small-Pox after Vaccination. | | |
|---|---|---|---|---|---|---|---|---|---|
| | Cases. | Deaths. | Per Cent. | Cases. | Deaths. | Per Cent. | Cases. | Deaths. | Per Cent. |
| Returns to Epidemiological Society........ | 2611 | 515 | 19·7 | 203 | 17 | 8·3 | 2013 | 59 | 2·9 |
| Edinburgh epidemic (1818-19) .......... | 205 | 50 | 24·3 | — | — | — | — | — | — |
| Norwich epidemic (1819) ................ | 200 | 46 | 23·0 | — | — | — | — | — | — |
| Hungerford (1838) .................... | 24 | 6 | 25 | — | — | — | 60 | 1 | 1·6 |
| Gateshead ........................... | 60 | 13 | 21·6 | 2 | 0 | — | 6 | 0 | — |
| Chelsea (1838-9)...................... | 161 | 45 | 27·9 | — | — | — | 27 | 1 | 3·7 |
| Wandsworth (1844-5) ................. | 58 | 16 | 27·5 | — | — | — | — | — | — |
| Glasgow Infirmary ..................... | 271 | 86 | 31·7 | — | — | — | 265 | 19 | 7·1 |
| Royal Military Asylum ................. | — | — | — | 12 | 4 | — | 27 | 0 | — |
| Copenhagen, five epidemics (1824-35) .... | 746 | 192 | 25·7 | — | — | — | 3093 | 66 | 2·1 |
| Wurtemburg (1831-6) ................. | — | — | — | — | — | — | 1055 | 75 | 7·1 |
| Vienna (1834)......................... | — | — | — | — | — | — | 200 | 16 | 8·0 |
| Malta ................................ | 4850 | 1022 | 21·07 | — | — | — | 2720 | 116 | 4·2 |
| Ceylon (1830) ......................... | 131 | 58 | 44·2 | 23 | 2 | — | 260 | 34 | 13 |
| Ceylon (1833-4) ........................ | 228 | 88 | 38·5 | 2 | 0 | — | 197 | 21 | 10·6 |
| Mauritius (1840-1)..................... | 281 | 120 | 42·7 | — | — | — | 421 | 30 | 7 |
| Small-Pox Hospital .................... | 2654 | 996 | 35·55* | 47 | 9 | 18.9* | 3094 | 268 | 6·76* |

* In calculating this percentage, the cases of death from superadded diseases have been subtracted, amounting, according to Mr. Marson, to 81 among the natural cases, 1 among the secondary, and 63 among the vaccinated.

as regards the Small Pox Hospital, cases of superadded disease have not been separated: thus of the fifty-nine* deaths reported in the first line, death was attributed by the reporters themselves, in several instances, to some superadded disease; in one case, to abdominal inflammation; in two to laryngitis; in one to pneumonia; in one to erysipelas; in one to scarlatina, immediately following the attack of small-pox (which last three cases all occurred in a hospital); and in one case it is stated also, that the deceased had suffered for years from chronic bronchitis. In none of these cases was the attack of small-pox itself of such a nature as would have led to a fatal result. If allowance were made, then, for these two circumstances—first, deaths included which were not properly attributable to small-pox; and, secondly, deaths in cases in which real evidence of efficient vaccination was wanting—the table would show a result still more favourable than it does to the modifying power of vaccination.

The ratio of mortality in the Small Pox Hospital is high, both as regards natural small-pox and small-pox after vaccination; much greater than prevails in communities at large even under epidemical influence, from circumstances which must be obvious. In the first place, the severer cases are those naturally sent to a hospital, the milder being kept at home; or if milder are presented when the hospital is full, the severer are taken in in preference: in the next place, there are hospital diseases, as gangrene, erysipelas, and the like, to which the patient would not have been subjected at home: and lastly, there is a hospital influence, arising from the collecting together patients all infected with such a disease as small-pox, and which, independently of the production of any special superadded disease, aggravates mortality. So it happens that, allowance being made for the superadded diseases, the mortality is still in advance of what occurs elsewhere. From the 268 deaths occurring after vaccination, Mr. Marson calculates that sixty-three should be deducted on account of superadded disease, and the rate per cent. of mortality then remaining will be 6.76 per cent.: and I must now direct attention to his masterly analysis of this mortality, constituting, as it does, the most valuable contribution which has ever been made to this branch of the subject. While the mortality, then, in all reputed to have been vacci-

* These were *all* the deaths from small-pox after vaccination, which had been seen by a hundred and seventy-one medical men who had kept records; and there were many more, who had no numerical record, but who stated to the Society that they had never seen a death from small-pox after vaccination.

nated was 6.76 per cent., in those who were *merely stated* to have been vaccinated, but had no mark to show, it was 21.73 per cent.: and it is important to remark, that the statement which was relied on here was always a clear one, either from the patient's own recollection, or from the account of his or her friends, and their belief that it had taken effect properly. In those with cicatrices it was as follows:

| | | Mortality Per Cent. | Mortality Per Cent. |
|---|---|---|---|
| One vaccine cicatrix: | Well marked - | 4·13 | 7·57 |
| " " | Badly marked - | 11·95 | |
| Two vaccine cicatrices: | Well marked - | 2·68 | 4·13 |
| " " | Badly marked - | 7·29 | |
| Three vaccine cicatrices: | Well marked - | 1·63 | 1·85 |
| " " | Badly marked - | 2·32 | |
| Four vaccine cicatrices: | Well marked - | 0·99 | 0·74 |
| " " | Badly marked - | 0·00 | |

| | | |
|---|---|---|
| Total mortality: | With good cicatrices - - - | 3·04 |
| " | With bad cicatrices - - - | 9·77 |
| " | With one and two cicatrices - - | 6·21 |
| " | With three, four, and more cicatrices - | 1·30 |

This table is full of instruction. In the first place, it shows us how many there are who consider themselves vaccinated, and in whom lymph has undoubtedly been inserted (and in most instances, probably, with some local result), and yet who have next to no protection at all. They have been vaccinated in the popular sense of the term, *but they have never gone through the vaccine disease:* and of such Jenner held and taught that they were unprotected; and yet cases and deaths, such as these, are made to swell the lists of failures of vaccination. In the same way, in the epidemic of Ceylon, in 1830, out of 64 cases with no marks there were 18 deaths; of 69 with bad marks, unlike those of proper vaccination, there were 15 deaths; but of 127 with characteristic marks, but one death. In the same way again, in the epidemic of 1833-4, in 83 cases with no marks, or bad marks, there were 19 deaths; but in 111 with good marks, but two deaths; and the same point is abundantly illustrated in the returns to the Epidemiological Society. A mere statement, then, of vaccination, however apparently clear, independently of any mark, or with very bad marks, is not much to be relied on.

It shows, in the next place, a melancholy truth,—that there is much bad and inefficient vaccination.

And lastly, it exhibits, in a most striking way, the wonderful modifying power of thoroughly good and efficient vaccination,—the mortality in those vaccinated, and having four cicatrices, being less than one per cent. under the same circumstances, under which the mortality from natural small-pox is above 35 per cent.*

Now, it is only by taking into consideration both the protecting and the modifying power of vaccination, that we can arrive at a true estimate of the value of the discovery. Whether, when all shall be protected, small-pox shall cease from amongst us, and literally be exterminated, as Jenner anticipated, is a question which can only *then* be solved conclusively: in the meanwhile, men will settle it, probably, much according to the constitution of their minds, though there are great facts bearing upon it, and leading to a strong inference regarding it, not to be overlooked. But that, as a plague and devastating disease, as we all know it once to have been, it must already be exterminated, or that *it must be by our own fault and negligence if it be not exterminated,* seems quite clear. The means are certainly within our hands; if the result has not been attained, we must have neglected them or misused them.

Before proceeding to illustrate this by a brief reference to the change which has been induced in the mortality of mankind from small-pox, since vaccination was introduced, let me be permitted to observe, that the facts I have brought forward seem clearly to establish that vaccination is greatly to be preferred to the old process of inoculation, as a protection against small-pox, so far even as the individual is concerned, and without reference to other considerations of still greater weight, to be mentioned immediately. Vaccination involves no risk to life, and it will be seen, in the first place, how small is the chance (taking the population throughout under ordinary circumstances) that a vaccinated person will take small-pox at all, and then how very small the chance that, having taken it, he would die. But the process of inoculation itself kills outright, some of its advocates say five, and some three, of every thousand submitted to it: and for the survivors it has yet to be proved, that the protection they enjoy is greater than that afforded by vaccination, provided this be duly and efficiently performed. Of 1000 children vaccinated in infancy, it would be an extravagant supposition that fifty should ever take small-pox afterwards; and of these fifty (that is to say of the thousand vaccinated) how many would die? Why, according to the

* Of the forty-four medical men, mentioned in an earlier part of this paper as having suffered from variola after vaccination, the disease was extremely mild in all but four—so mild, in some cases, that its real character is still a matter of doubt; and in two or three there was the variolous fever merely, and no eruption.

vaccination, as we generally have it, not more than one and a half: but if the vaccination be thorough, as Mr. Marson has shown, the deaths would be less than a third even of this number. That is to say, on the extravagant hypothesis that 5 per cent. of all vaccinated should take small-pox, there would not be more than one in 2000 vaccinated who would die of small-pox at any period of their lives; whilst of 2000 inoculated, six or ten might be expected to die, as the result of the operation itself.

I have endeavoured to deal with this question as one to be settled by accumulation of facts; yet the opinions of observers,—many of whom have had large opportunities of personal inquiry and observation on the points at issue,—are not to be overlooked, especially when they are found to agree with one another in a remarkable manner. It has happened, from circumstances, that communications from nearly two thousand medical practitioners in this kingdom, from medical men in Bengal, Bombay, Mauritius, the West Indies, and various other places, have passed through my hands within the last four years: there have been, as might have been expected from such a large number of individuals, various shades of opinion as to the extent and degree of modifying or of protecting power belonging to vaccination; there have been various complaints of the way in which vaccination is often carried out; suggestions for improvement; suggestions for extension; but in no instance a suggestion that we should give up vaccination and recur to inoculation. I do not remember half a dozen instances of a personal predilection for inoculation expressed; and the communications throughout indicate an extraordinary amount of confidence in vaccination, when properly performed; none, certainly, more so than the communications from the East and West Indies. I may, therefore, be allowed to express my astonishment at reading, some time ago, in a work* purporting to be a standard work of reference, that, "in the middle of the nineteenth century, the majority of the profession, in all latitudes and hemispheres, are doubtful as to the preponderance of advantages to be obtained from inoculation or from vaccination." Dr. Copland has been publicly challenged to state the grounds of this assertion, but has never done so. There are also other statements in the same work equally incautious, unfounded, and, as tending to check the diffusion of vaccination, pernicious; as for instance, that, as regards warm climates and dark races, vacci-

* COPLAND'S Medical Dictionary, vol. iii, p. 829.

nation has been demonstrated to be inefficacious, the correctness of which may be judged of by the returns from Ceylon and the Mauritius in the foregoing table: and again, that the law has made vaccination to supersede inoculation in warm climates and among the dark races as in this country, of which I can only say, that I hope to live to see the day when it may become true. At this present time, when the efforts of those who are striving to procure the much needed extension of vaccination are obstructed by so much idle clamour,—when such industry is manifested to revive and keep alive absurd prejudices, it is much to be lamented that any medical authority should have written with so slight a sense of the responsibility which attaches to every one who professes to be an instructor of mankind.

But while vaccination has incontestable advantages over inoculation, as regards the individual, there are other considerations which still more emphatically and urgently recommend the former practice, while they render the latter, independently of any legal prohibition, and under all circumstances where vaccine lymph is attainable, little short of criminal. The inoculated small-pox is capable, like the natural disease, of being imparted from individual to individual, and thus each inoculated person becomes a focus of contagion. In this way, during those times in which inoculation prevailed, the small-pox was constantly communicated. No fewer than twenty-three instances are given by correspondents of the Epidemiological Society, in which districts free from small-pox were infected, and the infection clearly traced to an inoculated case. This was previously to the prohibition of inoculation in England, and the good results of that prohibition are referred to by numerous correspondents. The same practice, though prohibited, goes on in Ireland at the present time with disastrous results;* and it is pointed out by the Commissioners of Inquiry appointed by the government in India, as one of the great causes of the diffusion of small-pox.† Hence it is that, during the period when inoculation was in vogue in England, there was no diminution, but rather an increase, in the mortality from small-pox; while, since the introduction of vaccination, there has been a diminution progressing year by year, as vaccination has become more and more diffused.

During the later half of the last century,‡ when inoculation

* Census for Ireland for 1851, part v, vol. i.

† Report of the Small-Pox Commissioners, etc. Calcutta: 1850.

‡ During the fifty preceding years, in which there was little inoculation, that practice having been introduced about 1720, and not become at all genewas practised, the mortality from small-pox, in proportion to the mortality from all causes, was for the

| | | Per 1000. |
|---|---|---|
| Ten years ending | 1760 - - - | 100 |
| „ „ | 1770 - - - | 108 |
| „ „ | 1780 - - - | 98 |
| „ „ | 1790 - - - | 87 |
| „ „ | 1800 - - - | 88 |

while the proportion subsequent to the introduction of vaccination was, for the

| | | Per 1000. |
|---|---|---|
| Ten years ending | 1810 - - - | 64 |
| „ „ | 1820 - - - | 42 |
| „ „ | 1830 - - - | 32 |
| „ „ | 1840 - - - | 23 |
| „ „ | 1850 - - - | 16 |

So also in foreign countries, the proportion of deaths from small-pox has fallen from sixty-five in the thousand, which it was during the period of inoculation, to seven in the thousand, which it is now, taking the average of a great variety of countries and a long series of years.†

The same fact may be illustrated in another way:—For the ten years ending 1800, the average annual mortality from small-pox, within the Bills of Mortality, on a population ascertained in 1801 to be 261,233, was 1780. Immediately on the introduction of vaccination, this mortality began to decline. When the Registrar-General's office was established in 1837, a thoroughly trustworthy source of information on the causes of mortality was opened ; and from this authority we find that the average annual deaths from small-pox in four years, ending 1841, on the Metropolitan population, ascertained in 1841 to be 1,948,369, were only 1659 ; and this includes the year 1838, a year of most remarkable small-pox mortality. In 1841 the Act was passed providing public vaccination in every Union, with the result, that the average annual deaths from small-pox in fourteen years, ending 1855, on the Metropolitan population, ascertained in 1851 to be 2,373,799, were further reduced to 821.

We thus have the following results :—

| | Population. | Aver. Annual Loss from Small-pox. |
|---|---|---|
| Ten years ending 1800 - - | 261,233 - - | 1780 |
| Four years ending 1841 - - | 1,948,369 - - | 1659 |
| Fourteen years ending 1855 - - | 2,373,799 - - | 821 |

rally received till the middle of the century, the average decennial ratio of deaths from small-pox to deaths from all causes, varied from 53 to 82 per 1000.

* *Vide* Report on the State of Small-Pox and Vaccination in England and Wales, and in other Countries, etc., by the Committee of the Epidemiological Society (Parliamentary Paper) 1853.

There are no records which enable me to state with accuracy the mortality from small-pox in the kingdom at large, previously to the establishment of the present system of registration; but, by distinct calculations, Dr. Lettsom and Sir G. Blane arrived at the conclusion, that the annual mortality in Great Britain and Ireland from small-pox was, during the last thirty years of last century, about 35,000: now I need not say that the whole population of Great Britain and Ireland was greatly less then than that of England and Wales is at this present time: yet the average annual mortality in England and Wales, for four years, 1838-41 inclusive, comprehending, as above, the very fatal year 1838, on a population ascertained in 1841 to be 15,914,148, was only 10,550: and, the Vaccination Act having been passed in 1841, the annual average mortality from small-pox, for seven years, 1847-53 inclusive, on a population ascertained in 1851 to be 17,922,768, was further reduced to 5,412.

These figures are extremely interesting, not only as showing the diminution of mortality from small-pox since the introduction of vaccination, but also as illustrating the value of legislative interference in diffusing the blessings of that practice.

But does this wonderful result exhibit all that vaccination is capable of effecting? If it did, we should indeed be a long way yet from the extermination of even fatal small-pox. The truth is, that of this annual aggregate of deaths, the immense majority are among those in whom vaccination has never been performed, and of whom we may safely say that, had it been performed, they would, so far as small-pox is concerned, have been alive still. Three-fourths, at least, of this mortality occurred under the age of five years: now, as death from small-pox in vaccinated children under five is of the rarest possible occurrence, there is no doubt that the whole of these, with scarcely any exception, had never been vaccinated. Of the remaining fourth, or about 1350 per ann., there are no data whatever which enable us to determine what proportion had, and what proportion had not, been vaccinated. But I do not conceive there can be any doubt that comparatively few of them had been vaccinated at all, and that still fewer had been well and efficiently vaccinated. Of 1091 deaths from small-pox, above the age of five, occurring in the course of sixteen years in the Small-pox Hospital, in only 191 was there clear evidence of vaccination, and in only thirteen out of these were there more than two cicatrices. Even of these thirteen, six died of superadded disease. I do not pretend to apply these facts rigorously to the

1350 deaths before us; but they give us some help towards a solution; and, taking them in connexion with what we have learnt above, concerning the powers of vaccination, I do not hesitate to express my belief, that the mortality now counted by thousands might speedily be reduced to hundreds, nay, even to tens, by the application of a vigorous and efficient system of public vaccination.

There is another way in which this subject may be illustrated. It is well known that there are few countries in Europe—of the countries from which I have seen returns there is not one—in which vaccination is so much neglected as, till within a recent period, it has been in this;* and the consequence is that, notwithstanding that the regulations of most of these States fall very short of a perfect system, yet in all of them the mortality from small-pox, as compared with the total mortality from all causes, is considerably less than with us. In England and Wales, on an average of the last seven years, and in London on an average of ten years, the mortality from small-pox has been fourteen out of every thousand deaths from all causes; while in Sweden, Bohemia, Venice, or Lombardy, it has not been above two.

The comparison is not honourable to our country. Yet the statement I have made presents us in the most favourable aspect, for it applies to England and Wales only, and does not take into account Scotland and Ireland, for the state of which countries our Government is equally responsible. Their condition, indeed, is almost incredible. In some towns in Scotland, as Greenock and Glasgow, the mortality from small-

---

* By an act passed in 1853, commonly called Lord Lyttelton's Act, it is now compulsory on the parents of all children born in England and Wales, to have them vaccinated within three months of birth. The working of this act has been most beneficial; the public vaccinations under one year of age, which were in 1851, 1852, and 1853, 186,539, 194,089, and 201,271, respectively, having risen in 1854 to 408,824, and in 1855 to 354,976; results which, having been attained without remonstrance or rebellion, show clearly, too, that there is nothing very obnoxious to the people in compulsory vaccination. In the face of such results, it may safely be affirmed that no minister would endeavour to repeal that act, and that no member of Parliament, endeavouring to do so, would meet with any success. The opponents of vaccination may safely be challenged to that issue. But there are many deficiencies in the act; and the whole administrative system, as regards vaccination, is faulty in the extreme. These deficiencies and faults are pointed out, and the requirements of a proper State provision for vaccination are suggested, in a memorial presented by the Epidemiological Society to the President of the Board of Health in 1855, and printed as a Parliamentary Paper. Much would have been done to remedy the defects existing, and to facilitate vaccination to the people, by the Government measure of last year. This was overlooked by the small and clamorous, but too successful, opposition, which opposed the Bill as a compulsory measure, but, by getting it withdrawn, left unattained all the advantages it would have secured, and compulsory vaccination still not one bit the less the law of the land.

pox has been, on an average of several years, 32 and 36, respectively, out of every 1000 deaths; and for all Ireland, during the ten years ending 1841, it was 49 per 1000, and in the province of Connaught, 60 per 1000 of the whole mortality.

The Census for Ireland, indeed, just published, for the ten years ending 1851, shows some diminution in this awful mortality; but the Commissioners expressly state their belief, that this is "in a measure owing to the general deficiency of returns for the early years over which this inquiry has extended." As it is, no less than 38,275 deaths are returned as having occurred from small-pox during this period, of which no fewer than 34,377 were under ten years of age. We may safely affirm that *the whole of these latter*, and by far the larger portion of the remainder, might have been saved by proper vaccination.

In Ireland, as I have already said, inoculation goes on. In Scotland there is no national system of vaccination. In England there is a system; and I have shown the happy results of the establishment of it, as well as the extent to which it falls short of accomplishing that which is required. Taking the state of vaccination in these kingdoms, and in the British empire generally, into account—the neglect of it, and its inefficient performance in too many instances,—and contrasting it with what may be done, and with what has been done elsewhere, I say, without hesitation, that it is a stigma upon us, and a foul disgrace, and one which must be swept away. The attention of the Legislature will soon be directed again to this subject, and it is one on which it behoves the Medical Profession to speak with no uncertain voice. If a thoroughly good system could be organised for this country, and shown to work, the extension of it to Scotland, Ireland, and the Colonies, might be looked for as a matter of course. To the establishment of this, then, let our efforts be directed; nor let the failures of the past discourage us: the cause of truth and humanity must ultimately prevail.

---

In publishing the foregoing essay in a periodical which is much read by non-medical men interested in questions of public health, it seems desirable to add a few words respecting eruptions, or other diseases, occurring at the time of, or immediately subsequent to, vaccination, on account of the great misapprehension which appears to exist on the subject, and of the many misstatements which have been made regarding it. These occurrences, when they have taken place, have been cited as evidence of disease communicated to the system through the medium of vaccine lymph, and have thus been made to support a prejudice which dates from the time when vaccination was objected to, because it infected the human system with the blood of a brute,—the awful consequences of which are related in pamphlets of the period, wherein we find that in one case "the face began to resemble that of an ox," while in another there were actually to be seen "patches of cow's hair."

In the first place, it is to be observed that the occurrence of any eruption or other disease along with vaccination, or so immediately succeeding it that it can with any plausibility be connected with it, is quite exceptional. The vaccine disease, under all ordinary circumstances, runs its own definite course; and when it has subsided, the system is left in every respect in the same condition in which it was before the vaccination was effected, save only that it is free from the liability to be infected by small-pox. This fact is really so certain and so familiar to all as to require neither proof nor illustration.

Secondly, it has never been pretended that any of the diseases that have occasionally been observed at the time of, or immediately subsequent to, vaccination have a special or peculiar character; they are the same diseases as are met with in children at all periods of their existence, as arising from cold, dietetic and other causes independent of vaccination, and *especially at the period of dentition, to which time vaccination is too often deferred.*

Thirdly, it is quite obvious that the mere occurrence of such eruptions or diseases as these at the time of, or immediately subsequent to, vaccination does not constitute any proof that they have been either caused by, or that their appearance has even been in any degree promoted by, the vaccination. To establish such proof it would be needful to show, on a number of persons sufficiently large to obviate any sources of error, that these eruptions are *more frequent* in children under the influence of vaccination, than they are in children of the same age, of similar constitution, and under all the same external conditions, who are not, or who have not recently been, subjected to vaccination. It is needless to say that no exact numerical inquiry of this kind has ever been made, nor does there seem the possibility of making it, —but the following observations, made on large numbers of individuals, seem to me to show, quite conclusively, that no such increased frequency does occur. Mr. Marson, who, since his residence at the Small Pox Hospital, has vaccinated upwards of 40,000 persons, states, "that he has never seen other diseases communicated with the vaccine disease, nor does he believe in the popular reports that they are ever so communicated. If such results were really true, as stated, and formed part of judiciously conducted vaccination, they must have come under the observation of your petitioner in vaccinating upwards of 40,000 persons."* Mr. Leese, who, in the course of his connection with the National Vaccine Establishment, has vaccinated an immense number, probably even a greater number than Mr. Marson himself, has never seen struma nor any rash of any kind produced. Mr. Marshall, late of Kington, Herefordshire, who, with his predecessor in practice, has kept an accurate register of all vaccinations performed by them from the year 1805 downwards, in 5,439 vaccinations never met with anything of the kind: and in a country district such as that in which Mr. Marshall practises, it is scarcely possible that any anomaly should have occurred without his being acquainted with it. The testimony of nearly the whole medical profession is to the same effect; and it is worthy of remark that the late Dr. Gregory, physician to the Small Pox Hospital, who was anything but an indiscriminate admirer of vaccination, did not think this question worthy of notice, but passed it by in total silence, in treating of vaccination in his work on the Practice of Physic.

There are, however, a few medical men, and they are comparatively very few, who state that, in particular cases, they have seen some eruptive or some strumous disease arising in the system so closely upon vaccination, as to excite in their minds the belief that it depended upon that process,—the vaccination, as they explain it, having acted as an irritant and roused into activity some latent constitutional peculiarity, as a cold might have done, or as the variolous inoculation, when it was practised, used in some instances to do, according to the testimony of many physicians of the last century. These gentlemen specially guard against the notion that these affections, which they associate with vaccination, are caused by the introduction into the system of any source of disease along with the lymph employed, by expressly mentioning in many instances that the same lymph used on other children developed no such affections: they

* *Vide* Mr. Marson's Petition to the House of Commons, in *Medical Times and Gazette*, August 30, 1856.

believe, in short, that the occurrence is due to the constitutional predisposition of the child vaccinated, and not to the source from which the lymph was taken. The explanation is no doubt correct *if the fact be established:* but with very great respect for those of my medical brethren who hold this opinion, I cannot but think, for reasons mentioned in the last paragraph, that what they have taken to be a consequence may have been mere coincidence, and that in constitutions such as they describe, the affections might have arisen in the same way and at the same time, had no vaccination at all been performed. But however this may be, the important practical point is, that the development of these affections is treated of as altogether an exceptional occurrence, and the fear of the possibility of inducing them has never influenced one of these gentlemen to withhold from any child the blessings of vaccination.

The foregoing facts yield, as we have seen, no support to the notion, that disease can be introduced into the system from without, through the medium of vaccine lymph. This hypothesis, indeed, is not a medical doctrine at all, but is the explanation of parents "unwilling", as Mr. Marson states, "to believe that there is anything constitutionally wrong in their offspring". All medical men well know that it is not possible to impregnate the system with any disease, except such as have a distinct and tangible materies morbi, like small-pox itself, psora, and syphilis, and then only by taking this materies morbi, and directly introducing it into the blood. Attempts to introduce diseases of other kinds—even the most contagious—have failed, though the greatest pains have been taken to secure the success of the experiment: and those who are aware that scrofulous pus has purposely been applied to the wounds made for vaccination, as well as to wounds made without any reference to vaccination, without the result of producing scrofula, can scarcely be made to believe that this contamination of the system could be effected by pure lymph taken from the arm of an apparently healthy child. For whether as regards strumous, or cutaneous disease, said by parents to be introduced into the system through the medium of the lymph employed, there is, generally, this peculiarity, that the child from whom the lymph was taken has not had the disease which he is said to impart.

In conclusion, I wish to make one or two observations on the extent to which this alleged fear of the introduction of other diseases really operates as an obstacle to the reception of vaccination by the poorer and more untaught classes. I am quite satisfied that this has been enormously exaggerated, principally from not discriminating between the real reason for leaving a thing undone, and the alleged reason or excuse for not having done it. That the real reason for the neglect of vaccination among the poor is apathy and indifference,—conditions of mind too common amongst those whose thoughts are engrossed in procuring bread for the day,—and that there is no deeply rooted prejudice against it amongst them, seem to me to be clearly proved by the fact that they always most readily and gladly embrace it, not only under fear of impending small-pox, but whenever their attention is specially called to it by the authorities,* or whenever extraordinary facilities are afforded for having the operation performed. There is a striking illustration of this in the first year's working of the Compulsory Vaccination Act. In that year, besides 408,824 children under one year of age vaccinated in conformity with the act, there were vaccinated 290,111 above that age, to whom therefore the act did not apply, and the vaccination of whom was entirely voluntary on the part of their parents. The explanation is clear: the parents, being obliged to bring their younger children, took the opportunity to bring also the others whose vaccination they had previously neglected. Negligence, then, is the real reason, but it is one which they are ashamed to acknowledge, and they therefore, if questioned, fall back upon the excuse we have been considering, as one more likely to command respect. But though the fear of contamination is not often, as I believe, a real reason for the *omission* of vaccination, it has a more real and a very beneficial influence when it resolves itself, as it generally does, into—to use their own frequent form of expression—" a fear of bad matter": an objection which never fails to give way, when they have the assurance that there has been proper care in selecting the individual from whom the lymph was taken. Such an assurance they have an undoubted right in every instance to require; and the desire for it is not the foolish prejudice which we have been combating, but a right and reasonable feeling, which, as leading to greater caution, should be encouraged, and is encouraged by every right-minded medical man. The use of impure lymph has injurious consequences of its own, though I do not believe that it can

* See Report of the Epidemiological Society on Small-Pox and Vaccination, 1853.

convey to one child the disease of another. It may act as an animal poison, producing erysipelas and allied disorders; or it may develope local irritation, or may go on to produce spurious vesicles, affording no protection, or but imperfect protection, against small-pox. But these are events which ought really never to occur: and that which it specially imports the public thoroughly to understand is, that they are not the results of the due performance of vaccination, but the result of its improper performance; that they are perfectly avoidable; and that they constitute, therefore, a reason for care in the performance of vaccination, but no objection whatever to the operation itself.

---

